# Doxorubicin delivery by pYEEIE peptide-functionalized rhodiola rosea-derived exosome-like nanovesicles for targeted melanoma therapy

**DOI:** 10.3389/fphar.2025.1619998

**Published:** 2025-07-28

**Authors:** Meitao Duan, Binbin Chen, Xue Yi, Ahmed Mahal, Linwei Song, Moxun Xu, Ahmad J. Obaidullah, Shuwei Yu, Chen Wang

**Affiliations:** ^1^ School of Pharmacy, Research Center for Sustained and Controlled Release Formulations, Xiamen Medical College, Xiamen, China; ^2^ Xiamen Xianyue Hospital, Xiamen Xianyue Hospital Affiliated with Xiamen Medical College, Fujian Psychiatric Center, Fujian Clinical Research Center for Mental Disorders, Xiamen, Fujian, China; ^3^ Key Laboratory of Functional and Clinical Translational Medicine, Fujian Province University, Xiamen Medical College, Xiamen, China; ^4^ Guangzhou HC Pharmaceutical Co., Ltd., Guangzhou, China; ^5^ Department of Pharmaceutical Chemistry, College of Pharmacy, King Saud University, Riyadh, Saudi Arabia

**Keywords:** rhodiola rosea, rhodiola-derived exosome-like nanovesicles, doxorubicin, pYEEIE peptide, drug delivery system, melanoma

## Abstract

**Introduction:**

Melanoma is the most common cause of skin cancer-related deaths due to its aggressive nature. Plant-derived exosome-like nanovesicles (PELNs) are promising natural nanoparticles for therapeutic applications owing to their biocompatibility and diverse bioactive components. However, research on Rhodiola rosea-derived exosome-like nanovesicles (RELNs) remains limited.

**Methods:**

This study evaluated the therapeutic efficacy and safety of a novel targeted drug delivery system, pYEEIE peptide-functionalized RELNs loaded with doxorubicin (DOX) (pYEEIE-RELNs-DOX), in melanoma-bearing mice.

**Results:**

Fluorescence imaging and histopathological assessments demonstrated that pYEEIE-RELNs-DOX exhibited superior tumor-targeting ability and significantly inhibited melanoma growth compared to free DOX and non-targeted RELNs-DOX. Importantly, pYEEIE-RELNs-DOX showed no toxicity to major organs (heart, liver, spleen, lungs, and kidneys), whereas free DOX induced cardiac tissue damage. Meanwhile, the serum ALT and AST levels remained normal, indicating no liver cell damage.

**Conclusion:**

These findings highlight the potential of pYEEIE-RELNs-DOX as a low-toxicity, high-efficacy targeted delivery system for melanoma therapy, providing a foundation for clinical translation.

## 1 Introduction

Melanoma is a tumor that develops from skin melanocytes (Naik, 2021) and has been the leading cause of skin cancer death due to its high invasion and lethality (Brehmer et al., 2012; Ci et al., 2019). Melanoma incidence has increased to varying degrees worldwide in recent years (Gulliver et al., 2022). Many western nations have high rates of melanoma worldwide, with Australia and New Zealand having the highest rates. This encourages pertinent melanoma research in these countries (Arnold et al., 2022). Melanoma is currently treated primarily with surgery, radiotherapy, chemotherapy, targeted therapy, and immunotherapy, among other techniques (Daud et al., 2010). Treatment efficacy is highly dependent on disease stage, with surgery being primary for early-stage disease and chemotherapy, targeted therapy, or immunotherapy used more for advanced stages (Wollina, 2022). Despite improved outcomes with immunotherapy/targeted therapy for melanoma, key limitations include late diagnosis, treatment-resistant advanced disease, cost/durability issues with novel agents, and chemotherapy’s constrained efficacy-toxicity profile. (Boutros et al., 2024). The quality of human life is seriously threatened by melanoma, and new technologies and approaches must be developed immediately to treat it effectively.

Plant-derived exosome-like nanovesicles (PELNs) are a diverse cell membrane-derived structure that typically ranges in size from 30 to 200 nm (Jackson et al., 2023) and contains natural nanoparticles containing bioactive compounds (Gupta et al., 2021; Van Niel et al., 2018). PELNs are commonly found in bodily fluids and contain proteins, lipids, and nucleic acids. They are currently thought to be key mediators of intercellular communication (Maacha et al., 2019; Nemati et al., 2022). It has intriguing biological action and may have a synergistic therapeutic impact with medicines (Fang and Liu, 2022; López de Las Hazas et al., 2023). PELNs are a developing study field with nanoscale structure, great safety, outstanding biocompatibility, and numerous sources. Based on these benefits, PELNs can be an effective drug delivery carrier (Madhan et al., 2024; Zhang et al., 2021). Furthermore, in addition to loading active components, PELNs can change their structure to achieve specific goals or exert multi-component synergy (Li et al., 2018; Manzari-Tavakoli et al., 2024). Rhodiola rosea is a perennial herb from the Crassulaceae family. It is mostly generated on the Qinghai Tibet Plateau, the Yunnan-Guizhou Plateau, and mountains above 2,500 m in northwest China. The roots and stems are utilized for medicinal purposes. Rhodiola rosea’s broad use in the pharmaceutical industry can be ascribed to its several actions, including antioxidant, depressive, antitumor, and neuroprotective properties (Amsterdam and Panossian, 2016; Nabavi et al., 2016). Rhodiola rosea has several chemical components, including flavonoids, glycosides, polysaccharides, and other active ingredients (Panossian et al., 2010). Salidroside, one of Rhodiola’s active components, has been extensively investigated. Rhodiola rosea-derived exosome-like nanovesicles (RELNs) are extracellular vehicles obtained from Rhodiola (Du et al., 2019; Li et al., 2019). Aside from inheriting the action of the active chemicals found in their parent plant, they provide benefits such as safety, non-toxicity, cheap cost, and the ability for mass manufacturing, establishing them as a natural and viable drug carrier (Mu et al., 2023). The pYEEIE is a peptide sequence that binds specifically to the SH2 (Src Homology 2) domain and can influence physiological processes such as cell proliferation, differentiation, and migration (Park et al., 2002). The DSPE-PEG-pYEEIE complex system can precisely target specific cells or tissues by harnessing the unique binding capability of pYEEIE to specific cell surface or intracellular proteins. This enables applications such as targeted drug administration and modulation of specific cellular functions (Remsberg et al., 2012).

A widespread and representative anthracycline medication (Aiken et al., 2009), doxorubicin (DOX) has broad-spectrum anticancer action and is essential in the treatment of cancer. It is frequently used to treat gliomas, melanoma, and breast cancer. By inserting itself into cellular DNA and starting a topoisomerase, DOX mostly disrupts the tertiary structure of DNA, which is how it exerts its anticancer effects (Benjanuwattra et al., 2019). However, due to DOX’s dose dependence and potential side effects during treatment, its clinical use remains limited (Sangweni et al., 2022). Cardiotoxicity is one of the most serious adverse reactions of DOX, which can cause irreversible damage to the patient’s myocardial cells during the treatment process (Belger et al., 2023). In order to reduce the adverse reactions caused by DOX in cancer treatment, researchers have proposed many solutions, and relying on drug carriers is one of the more feasible strategies. Wei et al. (2019) demonstrated significant anti-tumor activity both *in vivo* and *in vitro* in the treatment of osteosarcoma using extracellular delivery of doxorubicin derived from bone marrow mesenchymal stem cells. Moreover, they used exosomes loaded with doxorubicin (Exo-Dox), which significantly reduced the semi inhibitory concentration of Exo-Dox on tumor cells, demonstrating that Eox-Dox can selectively reach tumor cells and reduce cardiac toxicity.

**FIGURE 1:**
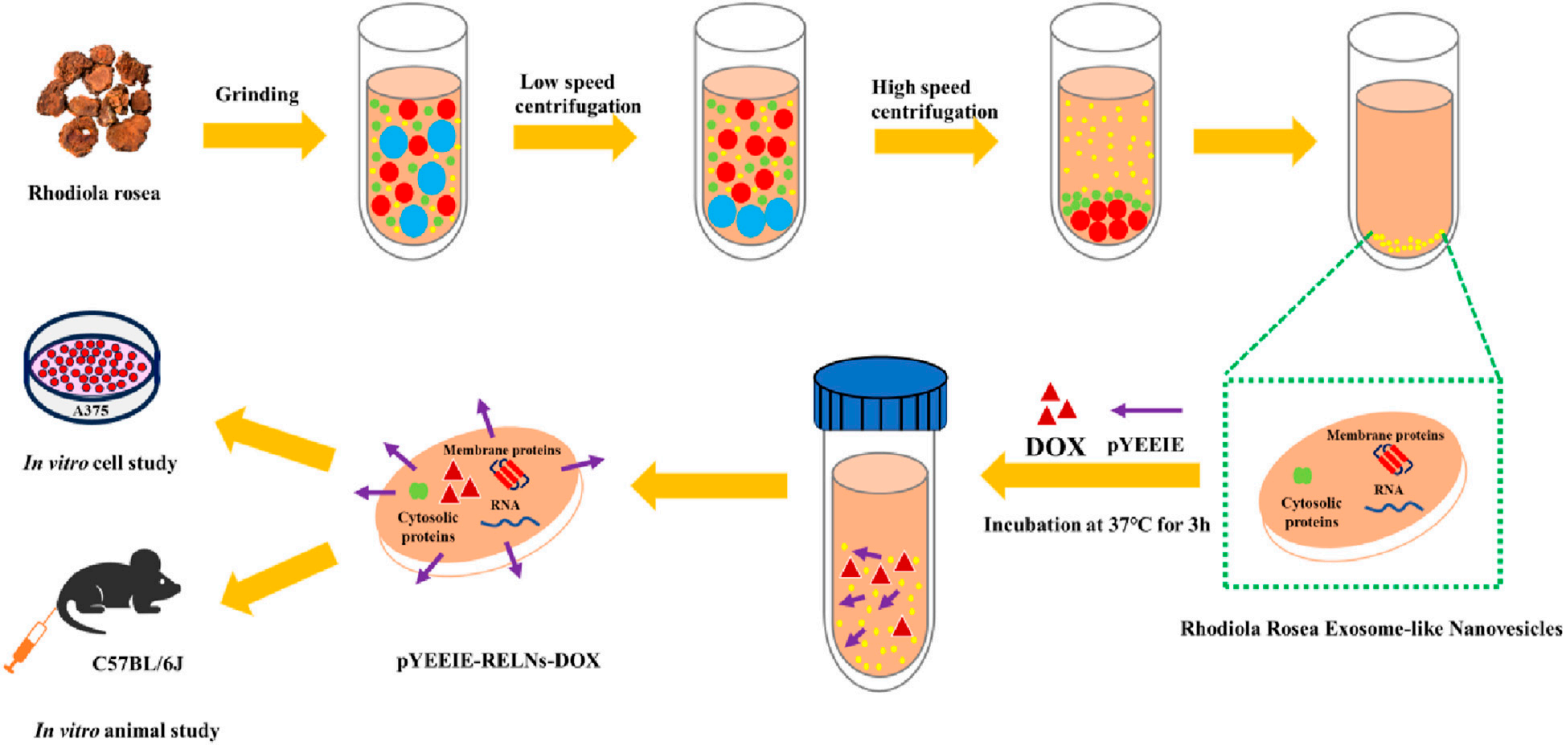
Design concept.

In our earlier investigation, we effectively created RELNs, examined their properties and activities, and built an efficient drug delivery system (pYEEIE-RELNs-DOX) that markedly reduced the proliferation of A375 cells. Further investigation into RELNs’ potential as a targeted drug carrier is made possible by their biological activity against melanoma. Further investigation into RELNs’ potential as a targeted drug carrier is made possible by their biological activity against melanoma. In this work, we concentrated on resolving the obstacles and problems that need to be resolved before we can completely advance the clinical usage of this drug delivery method. We evaluate its distribution and safety throughout the body for a low-toxicity, highly effective anti-tumor targeted delivery system with clinical translational potential and technical reserves for precision medicine (Figure 1).

## 2 Materials and methods

### 2.1 Materials

In Anhui (Anhui, China), rhodiola rosea was bought at the Bozhou Traditional Chinese Medicine Market. Dalian Meilun Biotechnology Co., Ltd. (Liaoning, China) supplied the DOX, Hematoxylin and Eosin (HE) staining kit. The supplier of DSPE-PEG-pYEEIE was Xi’an Ruixi Biotechnology Co., Ltd. (Xi’an, China). We bought C57BL/6J mice from Xiamen University’s Experimental Animal Center. Every animal experiment was carried out in compliance with guidelines that were authorized by Xiamen University’s ethics committee (XMULAC20240306031).

### 2.2 Isolation and identification of RELNs

After the RELNs were separated by differential centrifugation, they were gathered and centrifuged at high speed. The RELNs’ size, shape, and distribution were examined using transmission electron microscopy (TEM, Hitachi HT7700 EXALEN). The diluted RELNs’ particle size and quantity were measured using Nanoparticle Tracking Analysis (NTA, Particle Metrix/Zeta View). Additionally, after lysing the particles with RIPA lysis buffer, the protein concentration in the RELNs was ascertained using the BCA protein concentration assay kit. For precise methods, consult prior research (Xu et al., 2025).

### 2.3 Preparation and characterization of pYEEIE-RELNs-DOX

Ultrasound and co-incubation methods were used to develop the pYEEIE-RELNs-DOX targeted drug delivery system. Following this, 10% of the RELNs-DOX solution was mixed with pYEEIE powder, which was then incubated for an additional hour at 37°C to enable pYEEIE to alter the RELNs-DOX surface and give it targeting capabilities. The loading efficiency was computed. Furthermore, we employed infrared spectroscopy (IR) to ascertain the relationship between pYEEIE and RELNs. IR technology was used to prepare and evaluate freeze-dried samples of RELNs, pYEEIE, and pYEEIE-RELNs. For precise methods, consult prior research (Xu et al., 2025).

### 2.4 Construction and administration of melanoma model mice

Male C57BL/6J mice (6 weeks old) were acclimated for 1 week at 22°C–24°C before tumor inoculation. To establish melanoma models, B16-F10 cells were cultured to confluence, detached with 1 mL trypsin, neutralized with 3 mL medium, centrifuged (1,000 rpm, 5 min), washed twice with PBS, and resuspended to a density of 5 × 10^6^ cells/mL in PBS. After shaving the inoculation site, 200 μL of this suspension (containing 1 × 10^6^ cells) was injected subcutaneously into the right axilla of each mouse to ensure uniform cell distribution. Tumor growth was monitored daily until volumes reached ∼100 mm^3^ (typically 7 days post-inoculation), at which point treatment commenced. Mice were randomized into seven groups (n = 5): Control group, PBS group, RELNs group, pYEEIE RELNs group, DOX group, RELNs DOX group, and pYEEIE RELNs DOX group. All treatments were administered via tail vein injection every 48 h for seven total doses. During therapy, body weights and tumor dimensions (length/width) were measured every 48 h, with tumor volumes calculated as V = (Length × Width^2^)/2.

### 2.5 *In vivo* fluorescence imaging assay

Drug distribution in mice and injections into the tail vein was tracked using live imaging of small animals. It should be mentioned that as RELNs do not emit fluorescent signals, the mice in the pYEEIE-RELNs and RELNs groups must first be treated with DiO dye on RELNs before receiving DiO dye treatment. 8 h of fasting to prevent food from interfering with the fluorescent signal while recording. After hair removal, mice were anesthetized with isoflurane. Fluorescence distribution was monitored via *in vivo* imaging. Organs were subsequently excised for *ex vivo* imaging.

### 2.6 *In vivo* safety assessment of RELNs

Soaked the removed hearts, livers, spleens, lungs, kidneys, and tumors in tissue fixative after euthanizing the mice. Following organ collection, tissues were stained with hematoxylin and eosin (H&E). Acquired images of all tissue sections using microscopy. Before euthanizing the mice, blood was collected from the eye sockets. The collected mouse blood was left at room temperature for 30 min to naturally coagulate. Then, 1,000 g of the coagulated blood sample was centrifuged for 15 min. After centrifugation, the pale yellow serum located in the upper layer was obtained. The obtained upper layer serum was transferred to a clean EP tube to avoid absorption into the middle and lower layers. AST and ALT indicators were immediately detected. Use the AST and ALT assay kits to measure these two indicators, following the instructions. Use an enzyme-linked immunosorbent assay (ELISA) reader to measure the OD value and calculate the corresponding levels of AST and ALT in mouse serum.

### 2.7 Data analysis

To identify differences between groups, the experiment’s data were evaluated using unpaired *t*-tests or one-way analysis of variance (GraphPad Prism 9.5). When *p* < 0.05, the results were deemed statistically significant. Data presented as mean ± SD with 95% confidence intervals.

## 3 Results and discussion

### 3.1 Isolation and identification of RELNs

**FIGURE 2:**
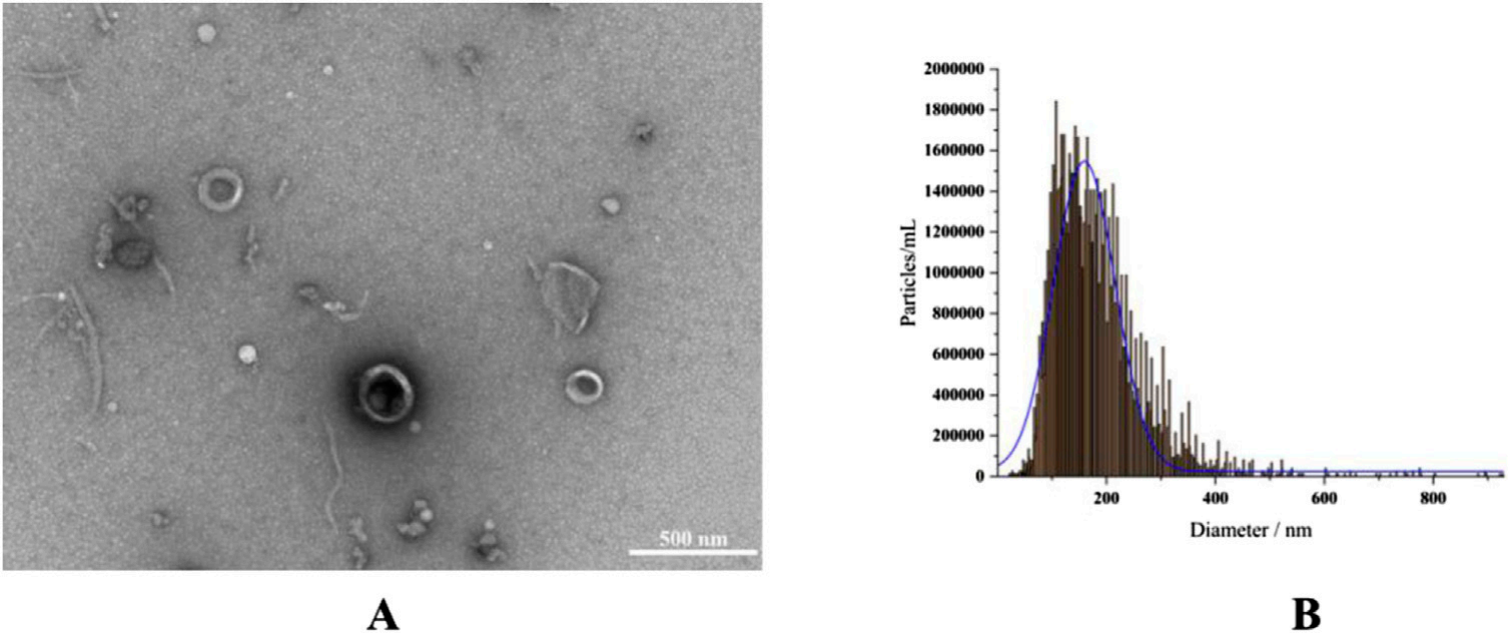
RELN identification and isolation. **(A)** A 500 nm TEM scale was used to describe the morphology of RELNs. **(B)** NTA was used to measure the particle size distribution of RELNs.

RELNs were separated in this investigation using differential centrifugation, and TEM and NTA were utilized to evaluate their shape, particle size, and potential (Figure 2). The results indicated that RELNs could be efficiently separated and enriched by differential centrifugation, which would also remove fibers, big particles, and insoluble contaminants from the raw materials. RELNs were almost spherical in shape, had a tea tray-like structure, and had the usual characteristics of plant extracellular vesicles, according to TEM pictures. In the meantime, RELNs’ lipid bilayer membrane structure was evident, and its thickness was comparatively consistent (Figure 2A). In line with other RELN types in earlier research, the average particle size of RELNs as determined by NTA was 173.1 ± 9.63 nm, primarily spread between 100 and 200 nm (Figure 2B). For instance, prior research has found that the particle size of four plant (ginger, grape, lemon, and celery) EVs is in the range of 100–200 nm (Lu et al., 2023), while the average particle size of Pueraria lobata EVs was 150.7 ± 82.8 nm (Zhang et al., 2024). In the meantime, the RELNs sample’s Zeta potential was found to be −15.70 mV, suggesting that it was stable.

### 3.2 Pharmacodynamic evaluation of tumor growth in mice

**FIGURE 3:**
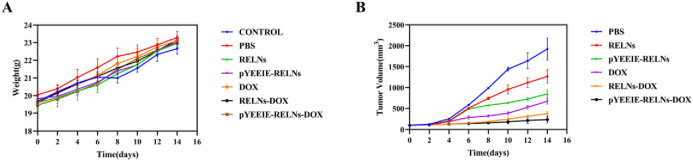
Body weight and tumor changes in mice. **(A)** Body weight changes in the mice (n = 5). **(B)** Tumor growth plots of the mice (n = 5).

Throughout the course of the treatment, the weight of each mouse group remained largely constant, and no appreciable weight loss was noted. Additionally, in the event of tumor suppression, the mice’s general physical health was maintained, and there was no discernible drop in weight brought on by tumor growth (Figure 3A).

All experimental groups exhibited progressive tumor growth. The PBS control group demonstrated the most rapid tumor expansion, reaching approximately 2000 mm^3^ by Day 14 of treatment. In compliance with animal ethics guidelines, all animals were humanely euthanized at this endpoint. During initial treatment phases, intergroup differences in tumor volume were statistically insignificant. However, with successive administrations, distinct therapeutic effects emerged: The pYEEIE-RELNs group showed significantly slower tumor progression than the RELNs group (*p* < 0.05). The pYEEIE-RELNs-DOX group exhibited markedly reduced growth rates compared to both RELNs-DOX and free DOX groups (*p* < 0.01). Critically, the pYEEIE-RELNs-DOX cohort displayed the slowest tumor growth kinetics, demonstrating superior antitumor efficacy among all treatment regimens (*p* < 0.001 vs controls). (Figure 3B).

**FIGURE 4:**
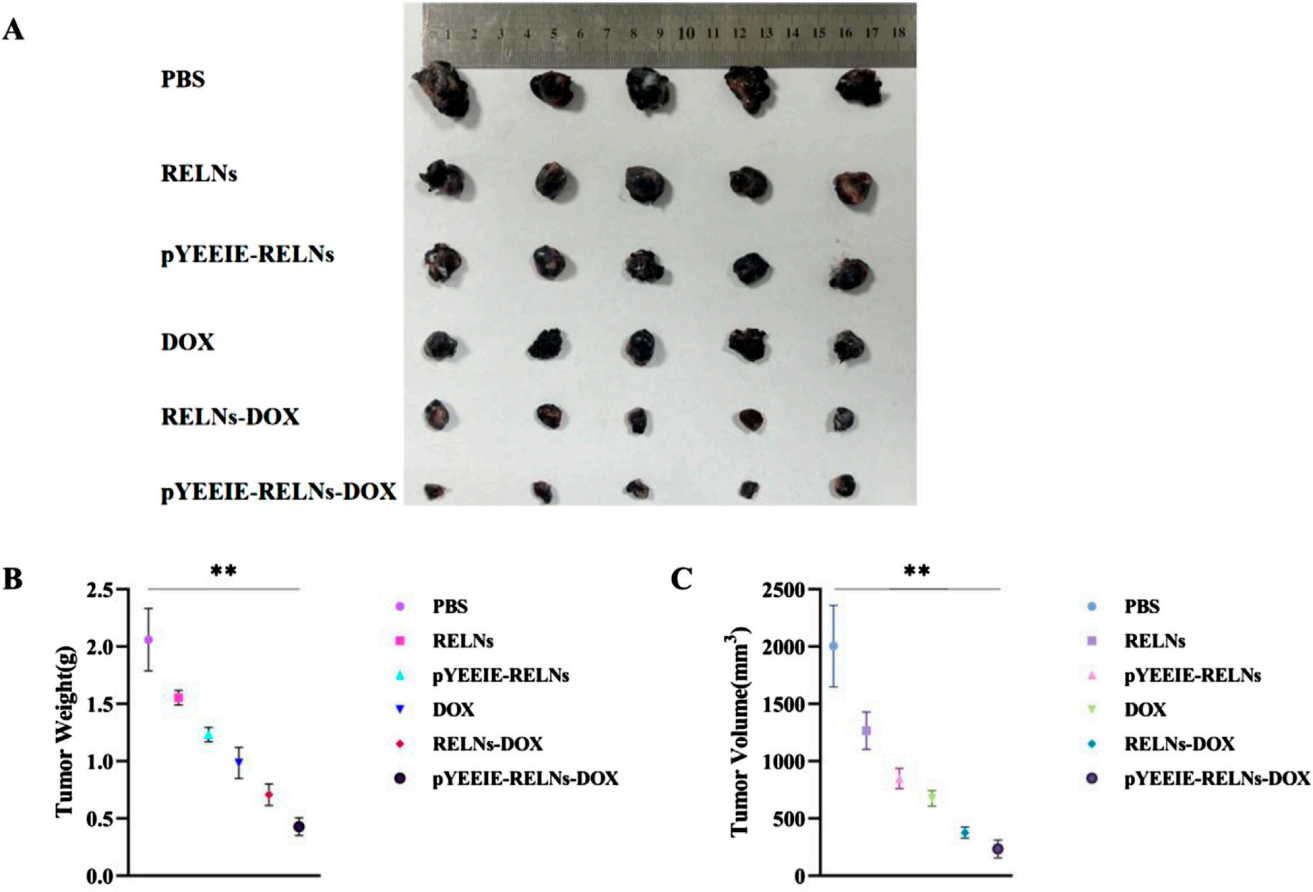
**(A)** Excised tumor photo of different groups (n = 5). **(B)** Tumor weight of mice (n = 5). **(C)** Tumor volume in mice (n = 5).

The PBS group did not have an inhibitory effect on tumor growth, while the other treatment groups showed varying degrees of therapeutic effects. Each group showed different levels of therapeutic effects when compared to the PBS group, there were notable variations in tumor volume and weight between the pYEEIE-RELNs-DOX group and the PBS group. (*p* < 0.01). Additionally, Statistical comparisons between DOX, RELNS-DOX, and pYEEIE-RELNS-DOX groupsrevealed that pYEElE-RELNs-DOX significantly reduced tumor weight compared to bothRELNS-DOX (*p* < 0.01) and free DOX (*p* < 0.01), while tumor volume differences becamesignificant from Day 9 onward (Figures 4B,C).

### 3.3 *In Vivo* distribution of RELNs

**FIGURE 5:**
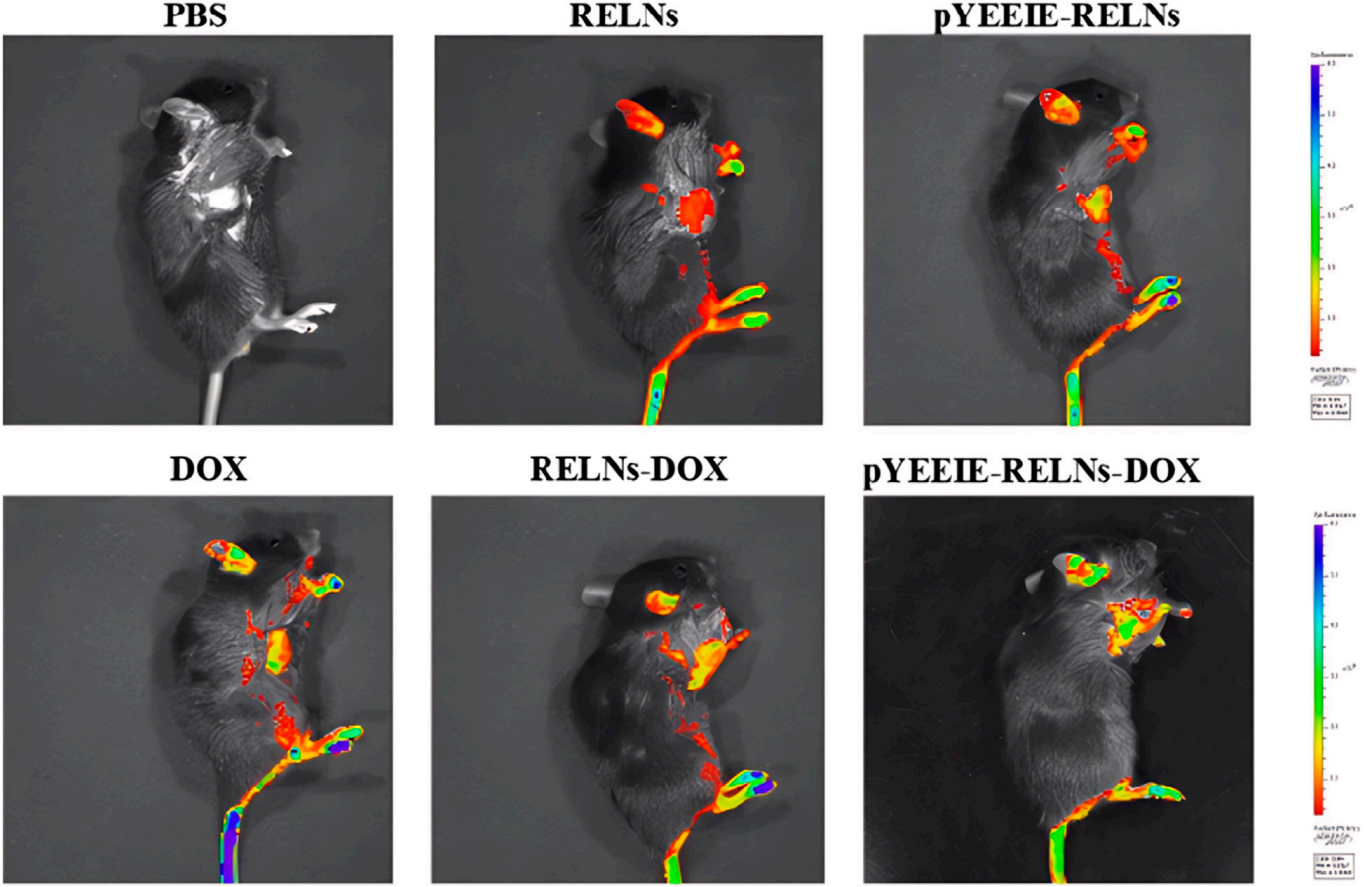
*In vivo* imaging of mice 24 h after administration.

The drug’s fluorescence signal was visible 24 h after administration, and the liver was where the majority of the tail vein injection’s fluorescence signal was located. Different treatment groups exhibited varying fluorescence distributions and intensities; the pYEEIE-RELNs-DOX group exhibited the greatest fluorescence labeling, suggesting the best enrichment effect in mice (Figure 5).

**FIGURE 6:**
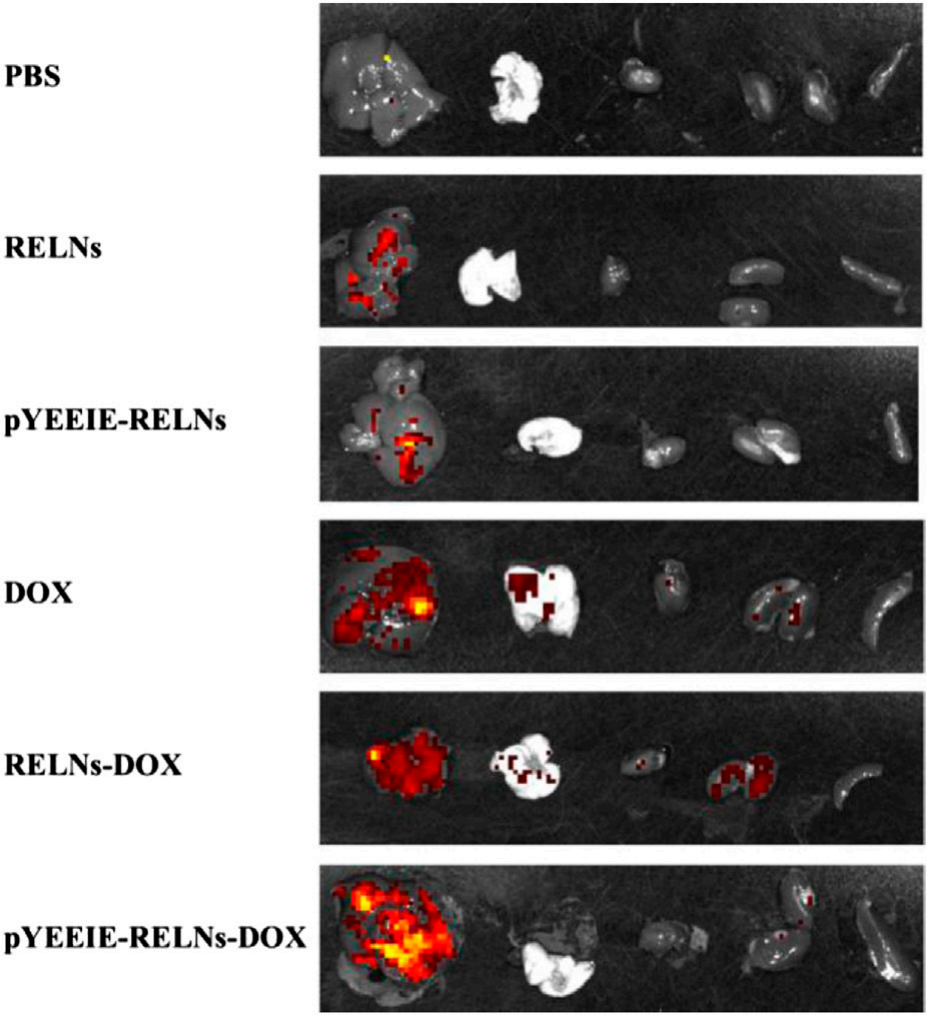
Fluorescence imaging of mouse organs 24 h after administration.

Simultaneously, when the mice were put to sleep, fluorescence imaging was done on the dissected organs, and the outcomes matched those of *in vivo* imaging of mice. Similarly, the pYEEIE-RELNs-DOX group exhibited the best enrichment effect, the best therapeutic efficacy, and the strongest and most concentrated fluorescence (Figure 6).

### 3.4 *In vivo* safety evaluation of RELNS through tissue section

**FIGURE 7:**
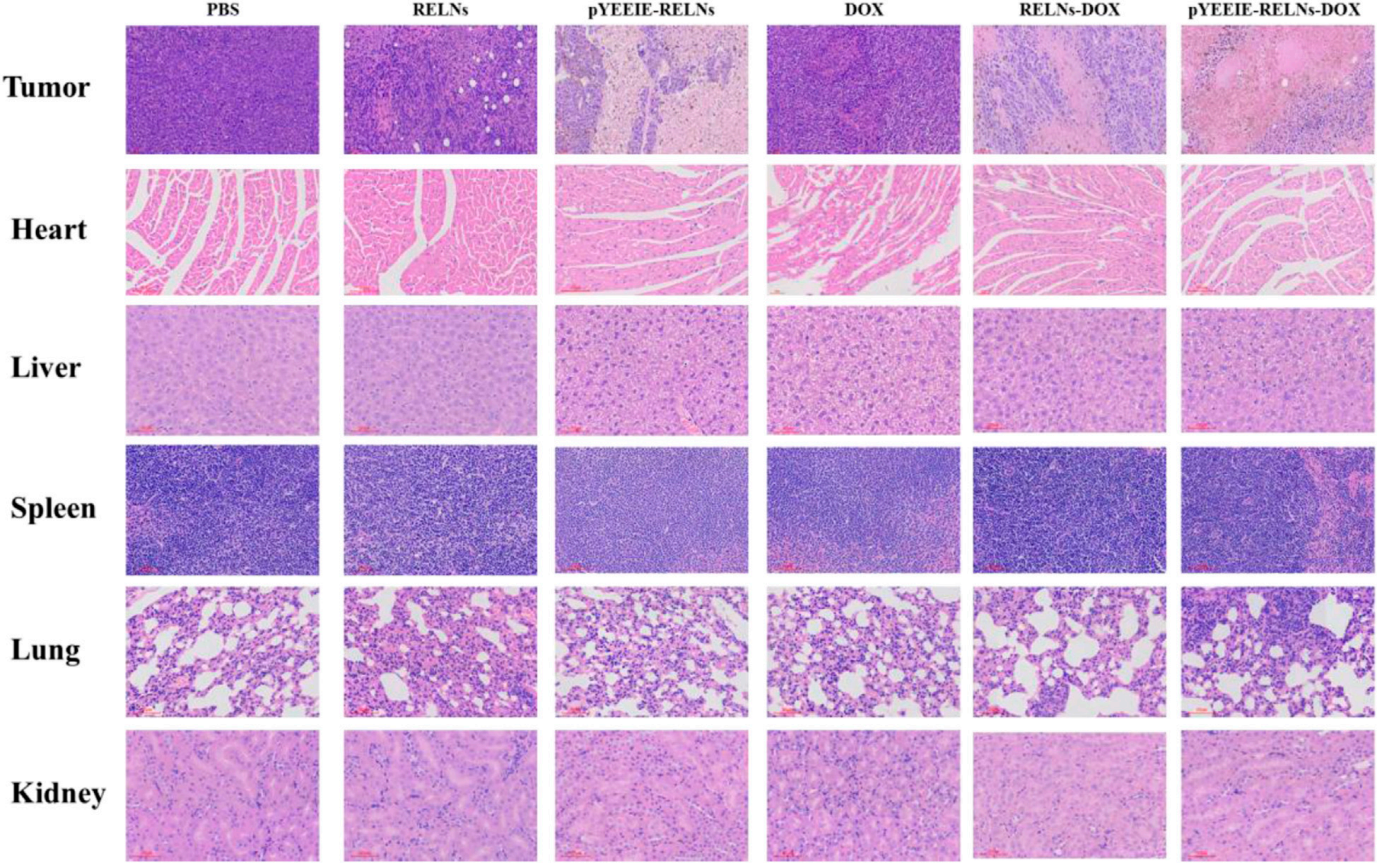
HE stained sections of the tumor, heart, liver, spleen, lung, and kidney. Scale bar: 60 μm.

The tissue slice results demonstrated that following HE staining, the PBS group’s tumor structure was essentially maintained normal, with many tumor cells packed closely together, distinct cell body outlines visible, and no discernible degeneration. The tumor in the DOX group was found to have modest damage, as evidenced by the presence of red coloring chemicals, nuclear fragmentation or disappearance, and necrosis of tumor cells in some regions. The RELNs-DOX group’s tumor damage was demonstrated to be worse, with greater necrosis, widespread necrosis and tumor cell degeneration, and nuclei that vanished. The greatest damage, the largest necrotic area, and the greatest amount of hemosiderin deposition were observed in the tumor tissue of pYEEIE-RELNs-DOX. Necrosis was evident in certain regions, and the extent of tumor damage in RELNs was similar to that in the DOX group. It was demonstrated that pYEEIE-RELNs had more necrotic areas and more severe tumor destruction than RELNs. In conclusion, the tumor tissue of pYEEIE-RELNs-DOX showed the most severe damage, indicating the best possible response to treatment. No discernible tissue or cell damage was found in the heart, liver, spleen, lungs, or kidneys of any of the therapy groups compared to the PBS group, indicating that the mice did not suffer any adverse side effects or toxicity while undergoing treatment. The aberrant cardiac tissue structure of the DOX group includes increased gaps, atrophy and degeneration of myocardial cells, sporadic rupture, and loose and disorderly organization of myocardial cells in some places. Nonetheless, the pYEEIE-RELNs-DOX group’s cardiac tissue structure displayed minor anomalies, including an ordered cell organization, modest cell atrophy in certain regions, and somewhat wider gaps. Showing that cardiac toxicity can be reduced by the pYEEIE-RELNs-DOX combination (Figure 7).

**FIGURE 8:**
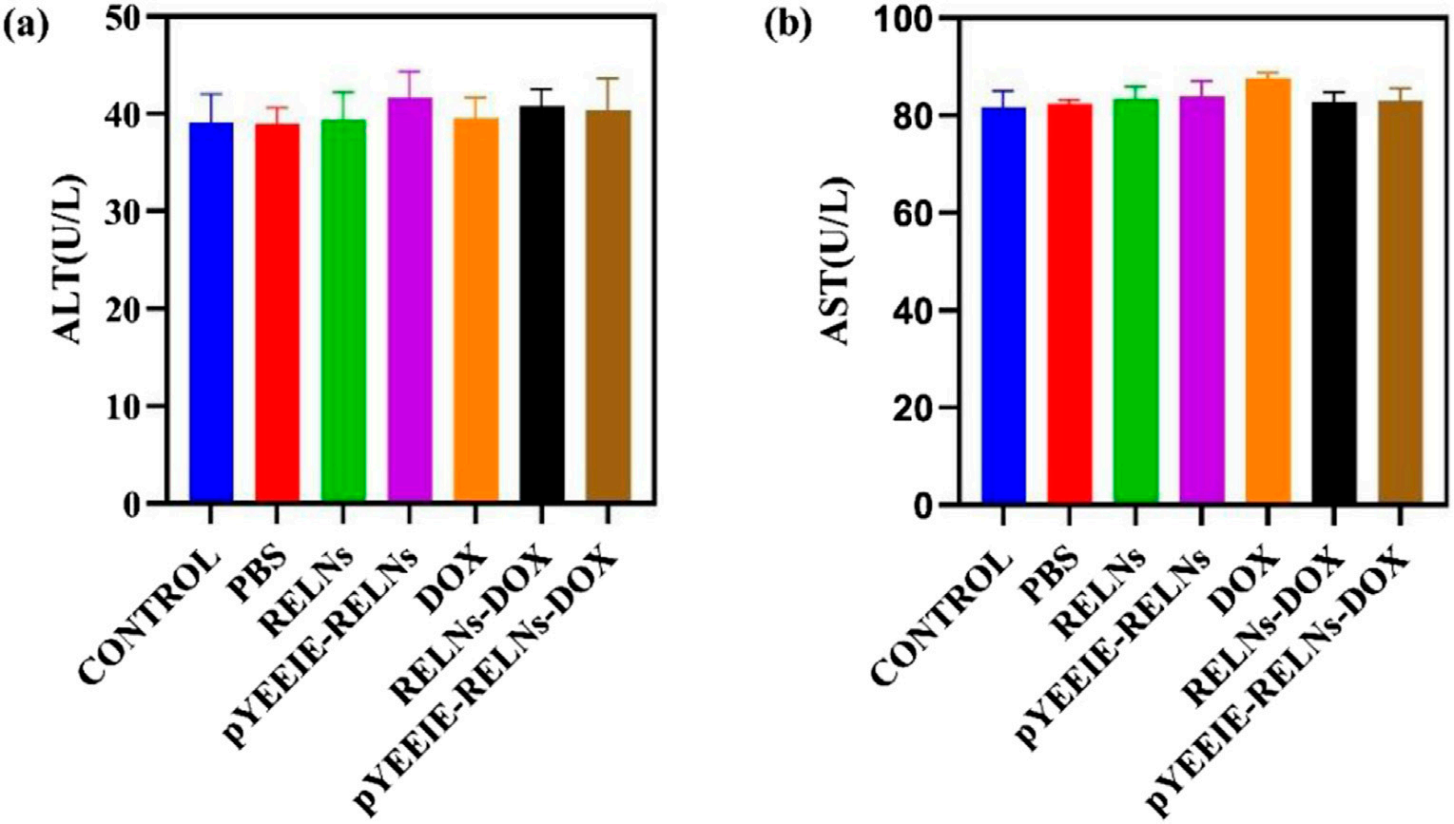
**(a)** The amount of AST present in the mouse serum (n = 5). **(b)** The amount of ALT present in the mouse serum (n = 5).

The values of ALT and AST sensitively reflect the degree of liver cell damage, and under normal circumstances, the levels of ALT and AST in the blood are relatively stable. If liver cells are damaged, these enzymes will be released into the bloodstream, causing their levels to increase. As shown in the figure, compared with the control group, the ALT and AST levels in the PBS group and each treatment group were similar and within the normal range. This indicates that under the current administration, there was no significant release of ALT and AST into the bloodstream, further suggesting that none of the groups caused significant damage to liver cells. (Figure 8).

## 4 Discussion

In this study, we successfully isolated and characterized RELNs using differential centrifugation. TEM and NTA analyses confirmed that RELNs exhibit typical plant-derived extracellular vesicle morphology with a spherical, cup-shaped structure and an average size of 173.1 ± 9.63 nm, consistent with other PELNs reported in the literature (Lu et al., 2023; Zhang et al., 2024). The observed zeta potential of −15.70 mV further aligns with the inherent stability of plant-derived nanovesicles (Zhang et al., 2021; Jackson et al., 2023), supporting their suitability as drug carriers.

The core finding of this work is the superior *in vivo* performance of the pYEEIE-RELNs-DOX. Our pharmacodynamic evaluation revealed that this targeted system significantly inhibited melanoma growth compared to free DOX and non-targeted RELNs-DOX, while simultaneously mitigating the cardiotoxicity hallmark of free DOX treatment. This enhanced therapeutic index, marked efficacy coupled with reduced toxicity, is a critical advancement. The significantly improved tumor accumulation demonstrated by fluorescence imaging directly correlates with the superior antitumor efficacy and strongly supports the role of the pYEEIE peptide in conferring active targeting capability. This aligns with the established principle that functionalization of nanocarriers with targeting ligands like peptides enhances site-specific drug delivery and efficacy (Remsberg et al., 2012; Li et al., 2018). Furthermore, the absence of significant histopathological damage in major organs (liver, spleen, lungs, kidneys) and normal serum AST, ALT levels across all RELN-treated groups underscores the biocompatibility and safety profile of Rhodiola-derived nanovesicles, consistent with safety reports for other PELNs (Madhan et al., 2024; Mu et al., 2023).

While the results are highly encouraging, several limitations of the current study must be acknowledged. Firstly, the sample size per group (n = 5) is relatively small, which may limit the statistical power for detecting more subtle differences between groups or rare adverse events. Secondly, the study focused primarily on phenotypic outcomes (tumor growth, organ toxicity, biodistribution). A deeper mechanistic understanding is needed, specifically, comprehensive assessment of inflammatory cytokines (IL-1β/6/8, TNF-α), ROS scavenging efficacy, and molecular profiling (qPCR/Western blot) will be incorporated to validate the anti-melanoma mechanisms of pYEEIE-RELNs-DOX. Thirdly, the potential impact of the hydration film on particle size measurement in PBS buffer, highlights a technical consideration for future characterization standardization. Finally, the study was conducted solely in a murine syngeneic model (B16-F10); evaluation in human melanoma xenograft models or models with acquired resistance would enhance clinical relevance. We fully acknowledge these limitations and will prioritize them in future work.

## Data Availability

The original contributions presented in the study are included in the article/supplementary material, further inquiries can be directed to the corresponding authors.
